# Dissolving Microneedles with Smart Design—A Tool for Enhancing Skin Permeation of Naltrexone Hydrochloride

**DOI:** 10.3390/molecules31122083

**Published:** 2026-06-13

**Authors:** Teodora Popova, Ivaylo Ganchev, Christina Voycheva

**Affiliations:** 1 Department of Pharmaceutical Technology and Biopharmaceutics, Faculty of Pharmacy, Medical University of Sofia, 1000 Sofia, Bulgaria; 2 Department of Pharmacy and Medical Cosmetics, Burgas State University, 8000 Burgas, Bulgaria; ivaylo-ganchev@uniburgas.bg

**Keywords:** dissolving microneedles, naltrexone hydrochloride, polyvinylpyrrolidone, polyvinyl alcohol, Poloxamer 407, transdermal delivery

## Abstract

Dissolving microneedles (DMN) could be considered as a minimally invasive alternative for transdermal delivery of naltrexone hydrochloride (NTX). In the present study, DMN patches with smart design were developed via a two-step micromoulding technique. The systems were composed of drug-free polyvinylpyrrolidone (PVP) and polyvinyl alcohol (PVA) blend microneedle tips, combined with a drug-loaded backing layer based on PVP and Poloxamer 407. The influence of polymer concentration in DMN tips and backing-layer composition on morphology, mechanical properties, drug release and permeation was evaluated. Mechanical studies revealed that intermediate polymer concentration (formulation MN-20%/2:1) provided superior structural integrity (13.57 ± 1.43% height reduction after compression) and efficient penetration up to the fourth Parafilm^®^ layer. Incorporation of NTX into the backing layer allowed for high drug loading, while a 2:1 PVP:P407 ratio provided higher toughness (1806 g/mm) as well as thermoresponsive and controlled drug release. In vitro permeation studies demonstrated significantly enhanced NTX delivery from DMN systems compared to simple matrix patches—an almost 4-fold increase in flux with 56% permeation of NTX up to 8 h. These findings highlight the importance of polymer composition in DMN design and demonstrate the potential of the developed systems as an effective platform for transdermal delivery of NTX.

## 1. Introduction

Naltrexone (NTX) is a synthetic μ-opioid receptor antagonist widely used in the treatment of alcohol and opioid dependence [1]. At low doses, NTX has also been explored experimentally for chronic pain, autoimmune, and neurodegenerative disorders [2,3]. Despite its clinical relevance, oral administration is limited by extensive first-pass metabolism, low and variable bioavailability (5–40%), short half-life, and dose-related adverse effects, all of which reduce therapeutic consistency [4,5,6,7,8]. Moreover, poor adherence to daily dosing regimens significantly reduces clinical benefit [9]. Modified-release subcutaneous [10] and injectable depot formulations were developed to address these challenges, providing sustained monthly exposure and improved adherence [9,11,12]. However, their use is limited by injection-site reactions and pain and requires medical supervision [13]. Transdermal delivery has been explored to overcome these limitations by bypassing hepatic first-pass metabolism and enabling more stable plasma concentrations [14]. In addition, it offers improved dosing convenience (e.g., daily or weekly application) and reduced systemic toxicity, optimizing naltrexone therapy [15,16,17,18].

However, conventional patches are restricted by the strong barrier function of the stratum corneum, particularly for hydrophilic drugs such as naltrexone hydrochloride [19]. Microneedle technology provides an effective strategy to overcome this barrier by creating transient microchannels in the skin, enabling minimally invasive delivery of active pharmaceutical ingredients (APIs) into the dermis, including compounds that are otherwise hindered by the skin’s low permeability [20].

The use of microneedles (MN) for transdermal delivery of NTX has been extensively investigated in preclinical research. A common approach involves a two-step procedure in which solid MN are first used to create microchannels in the stratum corneum, followed by application of a semi-solid formulation or conventional transdermal patch [21,22,23,24]. Although this method enhances NTX penetration compared to passive patch application, it imposes significant demands on patient compliance, as proper execution of both steps is required [25,26]. Moreover, the rapid in vivo closure of micropores limits the duration of drug permeation, thereby reducing the feasibility of sustained NTX delivery [24,27].

Dissolving microneedles (DMN) offer a single-step alternative by integrating drug delivery within water-soluble polymeric needles that simultaneously penetrate the skin barrier and release the encapsulated drug. This eliminates the need for additional patches or formulations, reduces patient burden, and avoids residual sharp waste [28]. However, their clinical translation is limited by restricted drug-loading capacity and mechanical considerations [29]. The amount of drug that can be loaded is often constrained by the mechanical strength required for successful skin penetration, and high drug loading can compromise needle integrity [30,31]. Moreover, the rapid dissolution of the needles in the skin may result in relatively short drug release, which can limit sustained delivery for therapeutics that require prolonged exposure [32,33].

To address these issues, smart strategies such as drug localization in the backing layer and incorporation of thermoresponsive polymers have been proposed to enhance loading capacity and enable controlled release. The backing layer, acting as a reservoir, permits substantially higher overall drug loading without compromising the mechanical robustness required for efficient skin penetration [30,32]. Thermoresponsive polymers such as poly(N-isopropylacrylamide) (PNIPAM) or poloxamers undergo reversible phase transitions in response to temperature changes, typically forming a gel at skin temperature [34,35,36,37]. This could delay micropores closure, ensuring longer, controlled, temperature-triggered and potentially on-demand release of the encapsulated drug [38].

The present study aimed to develop and characterize a dissolving microneedle system with an advanced architecture consisting of a PVP/PVA-based needle matrix and a thermoresponsive PVP–Poloxamer 407 backing layer loaded with naltrexone hydrochloride. The system was designed to improve mechanical performance, drug-loading capacity, and sustained transdermal delivery. A systematic formulation strategy was applied to evaluate the influence of polymer concentration in the microneedle tips and thermoresponsive components in the backing layer on overall system performance. NTX was selected as a model drug due to its poor permeability and well-recognized transdermal delivery challenges, providing a clinically relevant framework for assessing the potential.

## 2. Results and Discussion

### 2.1. Fourier Transform Infrared (FT-IR) Spectroscopy

**Figure 1:**
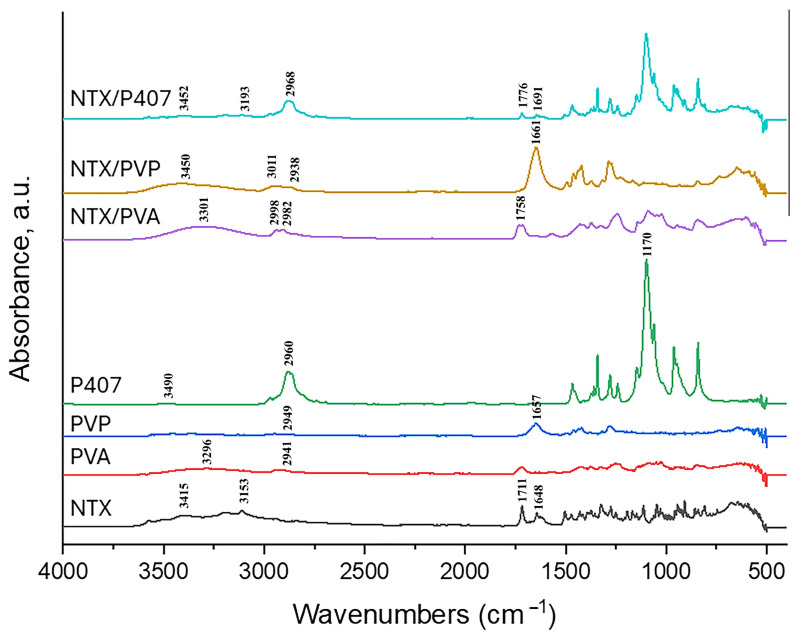
FT-IR spectra of naltrexone hydrochloride (NTX), individual excipients (polyvinyl alcohol, PVA; polyvinylpyrrolidone, PVP; and Poloxamer 407, P407), and their corresponding binary physical mixtures (NTX/PVA, NTX/PVP, and NTX/P407). The figure provides information about the potential drug–excipient interactions and compatibility within the formulation system.

The FT-IR spectra of the pure components and the corresponding API-excipient binary mixtures are presented in Figure 1. Naltrexone hydrochloride exhibited characteristic absorption bands corresponding to its molecular structure, including a broad band at 3100–3600 cm^−1^ attributed to O–H and N–H stretching, C–H stretching vibrations (aliphatic and aromatic groups) in the region of 3000–2850 cm^−1^, and a distinct peak at 1711 cm^−1^ assigned to C=O stretching of the ketone group. Additional bands observed between 1600 and 1500 cm^−1^ correspond to aromatic C=C vibrations. The observed spectrum is in good agreement with literature reports for naltrexone and its hydrochloride salt, confirming the presence of the expected functional groups [39,40].

Polyvinylpyrrolidone (PVP) showed a characteristic C=O stretching band of the pyrrolidone ring at approximately 1657 cm^−1^, along with C–H stretching vibrations at 2950–2850 cm^−1^ and bands related to C–N and C–O vibrations in the region of 1500–1000 cm^−1^ [41]. Polyvinyl alcohol (PVA) exhibited a broad O–H stretching band at 3200–3500 cm^−1^, C–H stretching vibrations near 2940–2900 cm^−1^, and characteristic C–O stretching bands in the region of 1220–1050 cm^−1^ [41,42].

Poloxamer 407 (P407) showed typical absorption bands corresponding to its poly(ethylene oxide)–poly(propylene oxide) structure, including O–H stretching vibrations at 3490 cm^−1^, C–H stretching at 2960 cm^−1^ and a prominent C–O–C stretching band at 1170 cm^−1^ [43,44].

Importantly, the FT-IR spectra of the binary mixtures did not reveal any significant shifts, disappearance, or appearance of new absorption bands compared to the individual components. This indicates the absence of chemical interactions between NTX and the investigated excipients, confirming their physicochemical compatibility within the developed formulations.

### 2.2. Preparation of Dissolving Microneedle Patches (DMN Patches) and Simple Matrix Patches (SP Patches)

The composition of the prepared DMN and SP patches is presented in Table 1.

Drug-loading capacity and mechanical considerations determined the decision for incorporation of naltrexone hydrochloride only into the microneedle backing layer rather than into the needle tips [30]. Dissolving microneedle tips have a small volume, which limits the amount of API that can be incorporated and the uniformity of the API content. Moreover, the direct inclusion of NTX into the tips may weaken the polymeric matrix, increase brittleness, and reduce insertion efficiency by disrupting intermolecular interactions within the polymer network. Localizing NTX in the backing layer could be used as a tool for providing higher and more uniform drug loading without compromising mechanical strength and penetration ability [45]. Following insertion and dissolution of the drug-free MN tips, the backing layer could act as a reservoir, enabling NTX diffusion through the formed microchannels and thereby enhancing transdermal delivery without affecting mechanical integrity.

Dissolving microneedle systems should fulfil several requirements to ensure effective transdermal delivery. They must exhibit a balanced combination of mechanical strength, insertion ability, and controlled dissolution [46]. Sufficient mechanical resistance is required to allow for the successful penetration of the stratum corneum without any bending or breakage, while maintaining structural integrity during handling and application. At the same time, the tips should not be too brittle, as this may cause fractures and incomplete drug delivery. Efficient penetration depends on a balance between stiffness and tip sharpness, enabling the formation of reproducible microchannels in the skin without causing pain. Besides this, after insertion, rapid and predictable dissolution of the microneedle tips within the viable epidermis is required for complete drug release.

The selection of the polymer blend (PVP and PVA) for the microneedle tips in the present study was based on their complementary physicochemical and mechanical properties. PVP is a highly water-soluble, biocompatible polymer that gives clear films and enables rapid dissolution and drug release following insertion. However, much of the literature data suggest that PVP alone may produce brittle structures with limited mechanical strength [47]. PVA alone is also generally not considered an optimal material for dissolving microneedle tips. Even though PVA contributes improved mechanical strength and flexibility due to its semi-crystalline structure and hydrogen-bonding capacity, enhancing resistance to deformation during insertion [47], it has slower dissolution and insufficient brittleness, which may compromise rapid needle dissolution and reliable mechanical performance during skin insertion [48,49]. The combination of PVP and PVA in a proper ratio has been shown to produce microneedles with better mechanical and dissolution performance than either polymer alone [47,49]. Both polymers exhibit good aqueous processability and compatibility, enabling homogeneous blending and a reproducible micromould casting procedure, and, at the same time, they are considered generally safe. The PVP/PVA blend is considered to ensure improved mechanical strength through intramolecular interactions (e.g., hydrogen bonding), providing a more robust network than either polymer alone [47,49]. Thus, the PVP/PVA blend appears to provide an optimized polymer matrix that meets the fundamental requirements for dissolving microneedles, namely sufficient mechanical integrity for effective penetration, controlled dissolution within the skin, and reliable manufacturing performance [48].

Considering the literature data as well as our preliminary studies, the design of the microneedle tips in the present study was optimized by varying the total concentration of the PVP:PVA polymer blend (10%, 20%, and 30% *w*/*w*), while the polymer ratio was kept constant at 2:1. Increasing polymer concentration is expected to improve mechanical strength and resistance to deformation during insertion. However, higher concentrations may also increase viscosity, compromise satisfactory mould filling, and slow dissolution. Thus, evaluating different polymer levels allows for the determination of an optimal balance between insertion efficiency and dissolution rate.

The backing layer plays a critical role in microneedle systems by serving as a reservoir for the API and supporting sustained delivery after skin penetration. In addition, it provides structural support to the microneedle array, allowing for adequate delivery. Thereby, its proper composition is of great importance for overall patch performance. The selection of Poloxamer 407 and PVP for the backing layer was based on their complementary physicochemical properties and their contribution to controlled drug delivery. PVP provides film-forming ability and structural stability, whereas Poloxamer 407 contributes flexibility and modulates hydration and drug diffusion. Poloxamer 407 is an amphiphilic triblock copolymer that exhibits thermosensitive behavior, undergoing sol–gel transition at approximately body temperature [44,50,51]. Following skin application, the hydrated backing layer containing poloxamer could partially gel, forming a more structured network [23]. The thermally induced gelation within the skin may help maintain the patency of the microchannels created by the dissolving microneedles, preventing their rapid closure and thereby prolonging drug diffusion. Moreover, the gel-like structure can function as a localized reservoir, sustaining NTX release and enhancing its permeation through the microchannels. By adjusting the PVP:Poloxamer 407 ratio (2:1 and 1:1) at a constant total polymer concentration (30%), it is possible to modulate matrix flexibility, hydration dynamics, and gel strength, which could affect drug release kinetics. Thus, the combination of PVP and Poloxamer 407 could be considered as a rational design strategy to obtain a mechanically stable, thermoresponsive backing layer capable of supporting prolonged and enhanced transdermal delivery following microneedle insertion.

**Figure 2:**
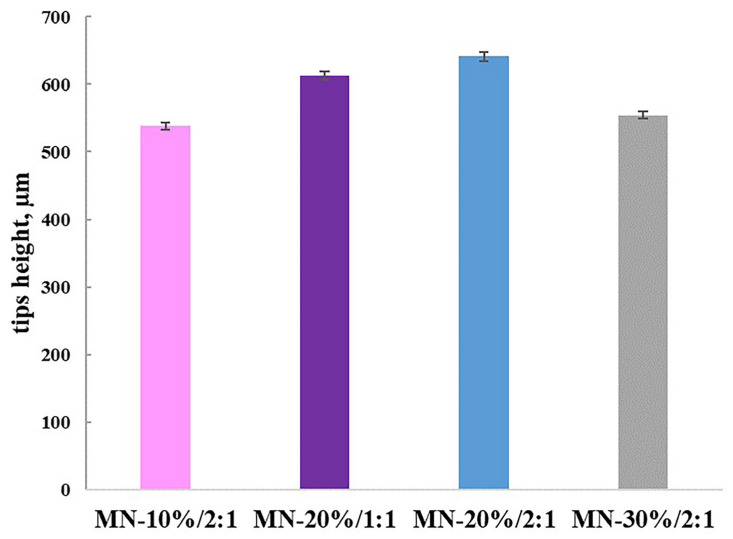
Tip height (µm) of the prepared dissolving microneedle (DMN) patch formulations, mean ± SD (*n* = 3).

**Figure 3:**
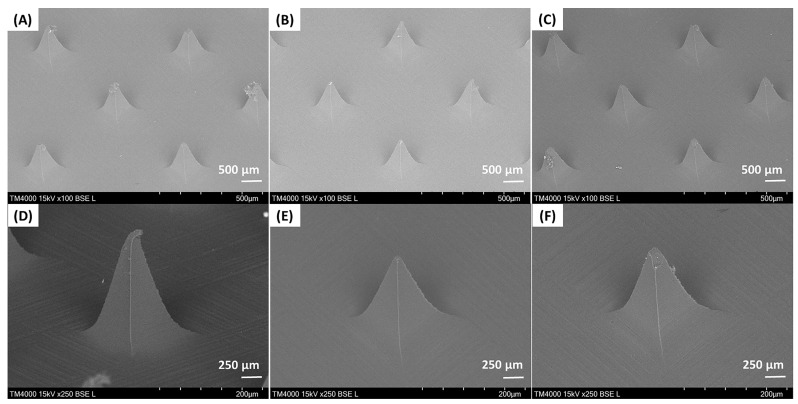
Scanning electron microscopy (SEM) images of dissolving microneedle (DMN) patch formulations MN-10%/2:1 (**A**), MN-20%/2:1 (**B**,**E**), and MN-30%/2:1 (**C**), illustrating morphological characteristics including the tip-curling phenomenon (**D**) and truncated tip formation (**F**).

The effectiveness of the microneedle fabrication process using the PDMS mould combined with centrifugal casting was verified by the determination of initial tip height via digital light microscopic examination (results presented in Figure 2) as well as SEM observation (Figure 3). Each mould consisted of 100 microneedle tips with an overall surface area of approximately 10 × 10 mm^2^. The produced microneedle arrays closely reproduced the dimensions and geometry of the mould cavities, exhibiting an average height of about 700 µm.

The initial height of the fabricated microneedle tips was expected to be close to the mould cavity depth of 700 µm. However, slight variations among formulations were anticipated due to differences in polymer concentration and solution viscosity. Since the geometry of the mould was identical for all formulations, deviations in needle height can primarily be attributed to polymer solid content, mould-filling efficiency during centrifugation, and shrinkage occurring during solvent evaporation [47]. The results showed that the final microneedle length (around 600 µm) was shorter than the original 700 µm master mould depth due to solvent evaporation, polymer network rearrangement, and compaction, which led to polymer shrinkage during drying. This phenomenon is typically observed in DMN production via micromoulding techniques based on hydrophilic polymers like PVP and PVA [48,52,53].

In addition, the results demonstrated a clear influence of polymer concentration and backing-layer composition on the final microneedle height. The MN-10%/2:1 formulation exhibited the lowest height (538 µm), which is consistent with the expected higher shrinkage associated with lower polymer solid content in the microneedle tips. The increased proportion of solvent in the 10% formulation likely led to greater volumetric contraction during drying, resulting in shorter final structures [47]. Both 20% formulations showed the highest microneedle heights, with MN-20%/1:1 reaching 623 µm and MN-20%/2:1 reaching 641 µm. These values suggested an optimal replication of the mould geometry at intermediate polymer concentration. The higher solid content compared to the 10% formulation likely reduced shrinkage while maintaining sufficient flowability during centrifugation, allowing for the effective filling of the mould cavities. The slightly greater height observed for the 2:1 backing-layer ratio may indicate subtle differences in structural support or drying behaviour, although the effect appeared modest. Interestingly, the MN-30%/2:1 formulation showed a lower height (574 µm) than both 20% systems, despite having the highest polymer concentration. This finding suggests that increased viscosity at 30% may have hindered complete cavity filling during centrifugation. Reduced flowability and potential air entrapment in the mould tips could have resulted in slightly shorter tips, offsetting the expected reduction in shrinkage [54]. Therefore, a balanced polymer concentration is required for reduced shrinkage, optimal viscosity and adequate cavity filling. Further SEM examination was performed in order to support the observation from the digital light microscope.

### 2.3. Scanning Electron Microscopy (SEM) of DMN Patches

SEM was used to study the micromorphology of the prepared DMN patch formulations containing 10%, 20%, or 30% polymer concentration. As can be seen from the SEM images in Figure 3A–C, all formulations possessed pyramidal tips with a smooth surface. A small number of shortened, truncated or blunted tips or arrays with tip-curling phenomenon were observed in the SEM images of formulations with 10% and 30% polymer concentrations (Figure 3D,F). The reduction in microneedle tip heights could be explained by different mechanical factors affecting mould filling, structural integrity, and drying behaviour [55].

Formulations at both the lowest and highest polymer concentrations (MN-10%/2:1 and MN-30%/2:1) produced noticeably shorter MN tips. At low polymer concentrations (10%), the formulation typically has low viscosity and reduced solid content. This can lead to incomplete filling of the micromoulds, especially at the tip regions, due to insufficient capillary force and poor retention of material within the cavities. In addition, during drying, the high water content leads to significant solvent evaporation, resulting in pronounced volumetric shrinkage and collapse of the polymer matrix. The low mechanical strength of the resulting structure further contributes to deformation or partial flattening of the microneedle tips, leading to reduced height [55]. A tip-curling phenomenon, observed for some of the MN tips of this formulation, could be attributed to greater overall shrinkage, faster and more uneven solvent evaporation, and lower mechanical stiffness of the partially formed microneedles [56]. In the early stages of drying, when the structure is still soft, it is more susceptible to deformation [57,58]. A higher polymer concentration, used in formulation MN-30%/2:1, resulted in extremely high solution viscosity, which could hinder efficient mould filling [59]. The increased resistance to flow prevents the formulation from fully penetrating the narrow tip regions of the mould, resulting in shorter or blunted microneedles [60,61]. At an intermediate (20%) polymer concentration, as in the MN-20%/2:1 formulation, a balance between viscosity, mould filling, and mechanical integrity was probably achieved, allowing for complete cavity filling and uniform solidification. This resulted in well-formed microneedles with optimal height and morphology, as observed in the SEM images (Figure 3E).

### 2.4. The Assay of API

The API content in the prepared patches (DMN and SP) was determined in order to evaluate the accuracy of the formulation process and the uniformity of drug incorporation within the polymer matrix. The results (Table 2) confirmed that all formulations contained NTX close to the theoretical values, indicating good reproducibility of the fabrication method and satisfactory content uniformity, thereby complying with the requirements for transdermal dosage forms.

Assessment of drug content is essential for microneedle systems, since they offer a limited volume of individual microneedle tips, hindering the uniform drug loading [29,62]. In many dissolving microneedle formulations where the API is incorporated directly into the needles, variations in filling efficiency and incomplete mould cavity filling may lead to heterogeneity in drug distribution [63]. In the present study, NTX was incorporated only into the backing layer. This design provided several advantages, considering formulation accuracy and dose uniformity. Since the backing layer accounted for the largest volume fraction of the patch, drug incorporation into this component enabled a more homogeneous distribution of the API throughout the matrix. As a result, the risk of dose variability associated with the limited capacity of microneedle tips was minimized. Moreover, localization of the drug within the backing layer allows the microneedles to act primarily as structural elements responsible for the creation of microchannels across the skin, while the backing layer functions as a drug reservoir, releasing the API through the formed diffusion pathways.

The satisfactory drug content results obtained for all formulations, therefore, confirm both the reliability of the preparation method and the suitability of the selected design strategy. Incorporating NTX into the backing layer contributed to improved dose uniformity and provided a smart solution to the common challenges associated with drug loading in microneedle-based delivery systems.

### 2.5. In Vitro Gelation Temperature, Gelling Time and Rheology

The thermoresponsive behavior of the poloxamer-containing systems was investigated in order to predict their potential in situ behaviour after application of the microneedle patches [44]. Although the developed formulations are solid films, upon insertion and subsequent exposure to interstitial skin fluids, the polymer matrix is expected to hydrate and undergo structural transformation. In this context, evaluating gelation temperature and gelling time can provide useful information on the formation of a gel-like layer that may influence API diffusion and release kinetics.

The ability of Poloxamer 407 to undergo temperature-induced sol–gel transition is well documented and is attributed to the formation and packing of micelles at certain temperatures [64]. At lower temperatures, the polymer chains are hydrated and exist as unimers, while increasing temperature enables dehydration of the polypropylene oxide blocks, leading to micellization and subsequent gel formation [65]. This transition typically occurs in the range of skin surface temperature, making Poloxamer 407 applicable for transdermal delivery [44].

Table 3 contains data about the determined gelation temperature and gelling time of the prepared poloxamer solutions (preparation described in the Materials and Methods section). The obtained results demonstrated that all investigated formulations exhibited gelation temperatures within a narrow range close to physiological skin temperature. The gelation temperature of the NTX-containing PVP:Poloxamer 407 (2:1) system was 34.2 °C, while a lower value of 31.6 °C was observed for the 1:1 ratio formulation. The decrease in gelation temperature with increasing poloxamer content could be explained by the greater availability of amphiphilic chains, which facilitate micelle formation and promote earlier gelation [66]. The presence of a higher proportion of PVP appeared to shift the gelation temperature slightly upward, likely due to interference with micellar organization and increased system hydrophilicity [50]. A similar trend was observed for the API-free systems, with gelation temperatures of 33.9 °C and 30.8 °C for the 2:1 and 1:1 ratios, respectively. The inclusion of naltrexone hydrochloride resulted in a slight increase in gelation temperature for both compositions, suggesting a modest interaction between the drug and the polymeric network. This effect may be attributed to changes in solvent structure or weak interactions that prevent micelle aggregation, although the magnitude of the shift remained relatively small, indicating that the thermogelling behavior is largely preserved [67].

The gelation time measurements at 35 °C further supported these observations. The PVP:Poloxamer 407 (1:1) formulation exhibited significantly faster gelation (9.17 s) compared to the 2:1 system (17.53 s), which was consistent with its lower gelation temperature and higher poloxamer content. The API-free formulations followed the same trend, with slightly shorter gelation times (8.32 s and 15.97 s, respectively). The presence of NTX led to a modest increase in gelation time, again suggesting a minor delay in micelle packing and network formation.

**Figure 4:**
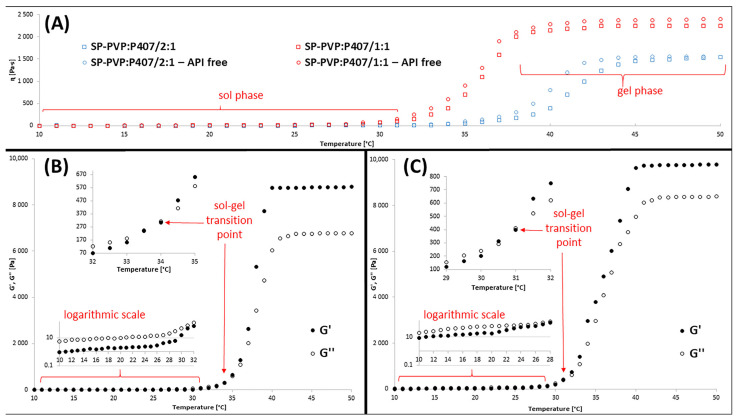
Temperature-dependent viscosity profiles of the prepared formulations (**A**), and storage (G′) and loss (G″) moduli as a function of temperature for SP-PVP:P407/2:1 (**B**) and SP-PVP:P407/1:1 (**C**) formulations. Insets with logarithmic scales corresponding to the lower-temperature regions, together with the G′/G″ crossover points, are included for improved visualization of the sol-to-gel transition behavior. Results are presented as mean ± SD (n = 3).

The rheological behavior of the prepared formulations was further evaluated to confirm the thermoresponsive properties of the Poloxamer 407-containing systems (Figure 4). The temperature-dependent viscosity profiles (Figure 4A) demonstrated a gradual increase in viscosity with increasing temperature, followed by a pronounced rise at temperatures approaching physiological conditions, indicating the transition from a low-viscosity sol state to a structured gel network. This behavior is characteristic of poloxamer-based thermosensitive systems and is associated with temperature-induced micellization and packing of polymeric micelles into a three-dimensional network structure [68,69].

The SP-PVP:P407/1:1 formulations exhibited earlier and more pronounced viscosity increases compared to the SP-PVP:P407/2:1 systems, suggesting stronger thermogelation behavior due to the higher relative content of Poloxamer 407. In contrast, formulations containing higher proportions of PVP showed weaker and broader transition profiles, likely due to the hydrophilic nature of PVP, which may interfere with micellar aggregation and reduce the sharpness of gel formation [70,71,72]. Incorporation of NTX slightly reduced viscosity compared to API-free formulations (* *p* < 0.05), suggesting partial interference of the hydrophilic drug with polymer–polymer interactions and micellar organization within the gel matrix [69,73,74]. Nevertheless, the formulations retained clear thermoresponsive behavior and maintained the ability to undergo gelation within a physiologically relevant temperature range, supporting their suitability for transdermal application.

Oscillatory rheological measurements further supported the thermoresponsive behavior of SP-PVP:P407/2:1 and SP-PVP:P407/1:1—Figure 4B,C, respectively. At lower temperatures, the loss modulus (G″) exceeded the storage modulus (G′), indicating predominance of viscous behavior characteristic of liquid-like systems. With increasing temperature, a crossover between G′ and G″ was observed, corresponding to the sol-to-gel transition temperature [71,72,75]. Above this transition region, G′ became higher than G″, confirming the formation of an elastic gel network. The observed rheological transition temperatures were consistent with the previously determined gelation temperatures and gelling times obtained using the tube inversion method, thereby validating the thermoresponsive characteristics of the prepared formulations through both qualitative and quantitative approaches.

The observed differences in rheological properties between the prepared formulations were found to be statistically significant (* *p* < 0.05 between NTX-containing formulations and API-free formulations and *** *p* < 0.001 between SP-PVP:P407/2:1 and SP-PVP:P407/1:1).

The solution containing a 30% polymer blend of PVP:PVA (2:1) was not expected to exhibit thermoresponsive gelation within the studied temperature range, as neither polymer possesses temperature-induced gelling properties.

Despite the differences between the formulations, all of them gelled at or near skin temperature and within a short time interval. This straightens the hypothesis that, upon hydration in the skin environment, a viscous gel layer can form. This behaviour is expected to contribute to controlled drug diffusion and may explain the sustained release and permeation profiles observed for the corresponding microneedle systems. Moreover, after MN tip dissolution, in situ gelled poloxamer could hinder micropore closure.

### 2.6. Mechanical Properties

#### 2.6.1. Three-Point Bend Testing of SP Patches

**Figure 5:**
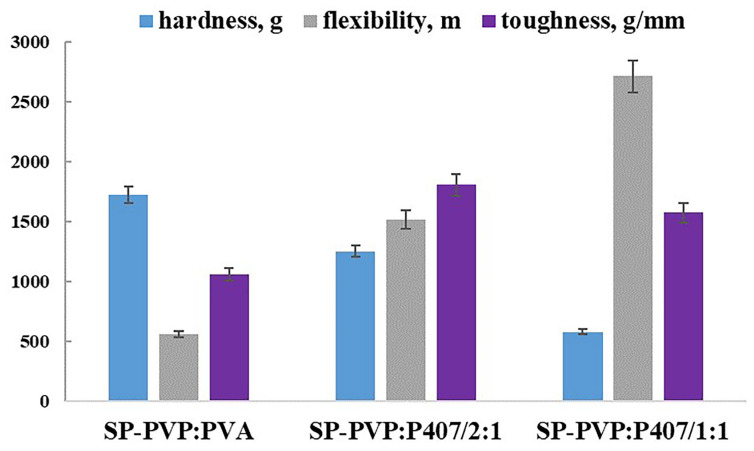
Mechanical properties of the prepared SP patch formulations expressed as hardness (g), flexibility (mm), and toughness (g/mm), presented as mean ± SD (n = 3).

The mechanical properties of the backing layer are critical to the overall performance of DMN patches. Beyond providing structural support for the microneedle tips, the backing layer helps ensure uniform force application during insertion, enabling efficient microneedle penetration into the skin. In addition, it affects patient handling, patch integrity during application, and, in systems where the drug is incorporated into the backing layer, it could also influence API release and diffusion behaviour. Therefore, the composition of the backing layer must be carefully optimized to achieve a balance between sufficient mechanical strength and flexibility [76]. Three-point bend testing was used to evaluate the mechanical properties of prepared simple matrix patches (Figure 5), corresponding to the backing-layer compositions, as it provides insight into their behaviour under deformation conditions similar to those encountered during patch application [77]. In this context, hardness reflects the maximum force required to deform the film and is indicative of its resistance to bending. Flexibility corresponds to the extent of deformation prior to fracture and represents the ability of the film to bend without breaking. Toughness describes the total energy required to induce failure and is derived from the area under the force–displacement curve, thus integrating both strength and flexibility.

Statistical analysis revealed significant differences in the mechanical properties among the prepared formulations (*** *p* < 0.001), which suggests that polymer composition strongly influenced the mechanical behavior of the prepared patches and may consequently affect handling characteristics and application performance. The formulation containing PVP:PVA (2:1) exhibited the highest hardness (1721 g), indicating a strong resistance to deformation. The combination of PVP and PVA provides a mechanically robust matrix, where the semi-crystalline nature of PVA contributes to increased hardness and structural strength through intermolecular hydrogen bonding [78,79,80,81,82]. PVP enables the formation of a homogeneous and dense polymer matrix due to its amorphous nature and strong intermolecular interactions [83]. However, without additional plasticisation, its relatively limited chain mobility may reduce flexibility (0.56 mm or 560 m), suggesting a relatively brittle structure with a reduced ability to accommodate deformation. Consequently, its toughness (1063 g/mm) was lower compared to the poloxamer-containing systems [84].

In contrast, the incorporation of Poloxamer 407 significantly modified the mechanical behaviour of the films. Both PVP:P407 formulations displayed reduced hardness but a marked increase in flexibility. This can be explained by the plasticizing effect of poloxamer, which reduces intermolecular interactions within the polymer matrix and enhances chain mobility [85,86,87]. The lowest hardness and highest flexibility value of PVP:P407 (1:1) formulation are indications of a highly deformable and soft material. The reduction in resistance to applied force limited the PVP:P407 (1:1) formulation’s overall mechanical robustness. Although the toughness (1574 g/mm) remained relatively high due to the large deformation before fracture, it was lower than that of the 2:1 formulation, suggesting that excessive plasticization compromised the balance between strength and flexibility [78].

Notably, formulation PVP:P407 (2:1) exhibited the highest toughness (1806 g/mm), suggesting an optimal balance between strength and deformability. The ability to sustain moderate force while undergoing substantial deformation results in improved energy absorption, which is desirable for maintaining integrity during handling and application [88].

Overall, the results indicated that polymer composition strongly governed the mechanical performance of the backing layers. While PVP and PVA contributed to rigidity and strength, poloxamer enhanced flexibility through its plasticizing effect [87,89]. Among the tested formulations, the PVP:P407 (2:1) system appeared to provide the most favourable combination of mechanical properties, combining sufficient hardness with enhanced flexibility and maximal toughness. This balance is particularly important for microneedle patch application, where the backing layer must be strong enough to support insertion while remaining flexible enough to conform to the skin without fracturing.

#### 2.6.2. Mechanical Strength of DMN Patches (Fracture Force Test)

Successful transdermal drug delivery using dissolving microneedle technology depends fundamentally on the ability of the MN tips to reliably pierce the stratum corneum, which represents the primary barrier of the skin. For consistent, reproducible insertion, MN arrays must be mechanically robust enough to withstand the compressive forces applied during manual or device-assisted administration. Insufficient mechanical strength may result in bending, blunting, or fracture of the needle shafts, ultimately compromising penetration efficiency and drug delivery performance [19,30,90].

The mechanical performance of dissolving polymeric MN patches is strongly influenced by the intrinsic properties of the polymers used, including polymer blend ratio, concentration, molecular weight, degree of crosslinking, and hydrogen-bonding interactions within the matrix. In addition, the incorporation of API or plasticizing excipients can significantly alter the rigidity, brittleness, and elasticity of the microneedles. Drug loading may disrupt intermolecular interactions within the polymer network, potentially reducing mechanical resistance, whereas certain excipients may either reinforce or plasticize the structure depending on their physicochemical characteristics.

For these reasons, mechanical characterization represents a critical preliminary step in the evaluation of MN systems before proceeding to ex vivo skin permeation or in vivo studies. Standardized mechanical testing provides insight into the resistance of the microneedles to compression and helps predict their likelihood of successful skin insertion under practical application conditions.

The mechanical strength of the DMN patch formulations was evaluated by measuring the reduction in needle height after application of a compressive force of 32 N. The initial height (H1) and post-compression height (H2) were determined microscopically, and the percentage reduction in height was calculated to assess structural deformation (Table 4).

The good overall appearance of all formulations could be attributed to their composition and successful fabrication method. The use of a PVA–PVP blend in microneedle preparation could enhance the mechanical performance of the formulation through intermolecular interactions between the two polymers. Specifically, hydrogen bonding occurs between the hydroxyl (-OH) groups of PVA and the carbonyl (-C=O) groups of PVP, resulting in a more cohesive, structurally stable microneedle matrix [91].

The results demonstrated a clear inverse relationship between polymer concentration in the microneedle tips and percentage height reduction after compression. The MN-10%/2:1 formulation exhibited the greatest reduction in height, decreasing from 538 µm to 267 µm, corresponding to a 50% reduction. This pronounced deformation indicated limited mechanical strength, which can be attributed to the low total polymer concentration (10%) in the microneedle tips. The lower solid content likely resulted in a less dense polymeric network, reducing resistance to compressive stress and increasing susceptibility to structural collapse. Increasing polymer content significantly improved mechanical performance. The higher solid content limited deformation under stress and increased robustness, likely due to reduced porosity, and enabled chain entanglement within the matrix. As a result, the height reduction in formulations with 20% and 30% polymer concentrations was below 17%. The lowest percentage reduction observed for MN-20%/2:1 suggested that this composition provided a more mechanically stable structure, potentially due to differences in polymer distribution or intermolecular interactions within the blend. Better mechanical performance of the backing layer of this composition (compared with the MN-20%/1:1 backing layer) could also contribute to the tips’ mechanical strength, since the backing layer supports insertion during application [92,93,94,95,96]. The observed differences in height reduction between the prepared formulations were found to be statistically significant (* *p* < 0.05).

#### 2.6.3. Parafilm Penetration Test of DMN Patches

Since the use of biological skin models introduces variability related to tissue heterogeneity and hydration state and possesses ethical restrictions, synthetic insertion models are frequently employed during early-stage testing. Parafilm^®^ M (supplied by Sigma Aldrich Chemie GmbH, Steinheim, Germany) is commonly used as a reproducible and convenient skin simulant due to its multilayered structure and uniform thickness. When folded into defined layers, it enables estimation of insertion depth by assessing the number of layers perforated under controlled loading conditions. Microscopic evaluation of the resulting microperforations enables quantitative assessment of penetration efficiency and comparative mechanical performance across different formulations, providing a practical, standardised approach for preliminary insertion studies [96].

The insertion ability of the DMN patch formulations was evaluated using a Parafilm^®^ M model, consisting of 8 eight layers, where the number of pores formed in each layer was counted as an indicator of penetration efficiency and depth. Since each Parafilm layer has an approximate thickness of 127 µm, penetration up to the fourth layer corresponds to an insertion depth of about 500 µm, which is generally considered sufficient to cross the stratum corneum and reach viable epidermal regions.

**Figure 6:**
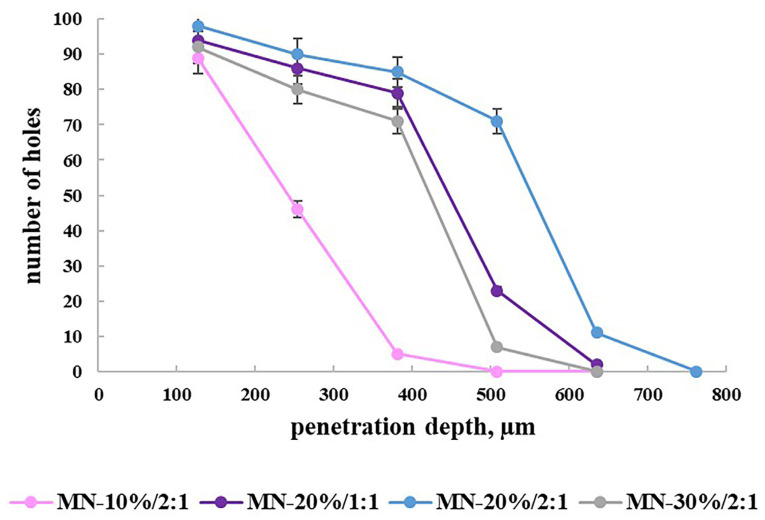
Parafilm M penetration test of the prepared dissolving microneedle (DMN) patch formulations, expressed as the number of microchannels (holes) formed per Parafilm layer, presented as mean ± SD (n = 3).

As can be seen from Figure 6, the MN-10%/2:1 formulation demonstrated limited insertion performance. Although 89 pores were observed in the first layer, the number decreased markedly in the second (46 pores) and third layers (5 pores), with no perforation detected in the fourth and fifth layers. This shallow penetration profile indicated insufficient mechanical strength to maintain structural integrity during insertion. These findings agree with the fracture force test, in which this formulation exhibited the greatest height reduction (50.37%) at 32 N, confirming substantial deformation under compressive stress. The high degree of structural collapse likely reduced the effective insertion depth.

In contrast, both 20% polymer formulations showed markedly improved penetration efficiency. MN-20%/1:1 produced 94, 86, 79, and 23 pores in layers one to four, respectively, with minimal penetration into the fifth layer. Similarly, MN-20%/2:1 generated 98, 90, 85, and 71 pores across the first four layers and 11 pores in the fifth layer. These results indicated consistent and deep insertion, reaching approximately 500–630 µm. The improved performance correlates well with the compression data, where these formulations exhibited good height reductions (17.61% and 13.57%, respectively), demonstrating sufficient mechanical robustness to resist deformation while maintaining adequate sharpness for efficient penetration. The superior performance of MN-20%/2:1, compared to MN-20%/1:1, in deeper layers is consistent with its lower percentage height reduction in the mechanical test and could be attributed to the higher toughness of its backing layer, enabling better mechanical support.

The MN-30%/2:1 formulation showed high pore numbers in the first three layers (92, 80, and 71), but a sharp decrease in the fourth layer (only 7 pores) and no penetration into the fifth layer. Although this formulation exhibited a sufficiently small height reduction in the fracture force test (15.34%), indicating good resistance to compression, its penetration depth did not exceed that of the 20% formulations. This suggests that excessive polymer concentration, while increasing stiffness, may compromise insertion efficiency. Increased viscosity during fabrication led to the formation of truncated and blunted tips, affecting their height and sharpness [97,98,99]. These findings support selecting the 20% PVA/PVP (2:1) polymer blend as the most promising configuration for achieving reliable skin insertion. Moreover, the optimal patch formulation complies with the requirements for painless application, since the tip’s height is enough to theoretically penetrate through the outer skin layers and reach the dermis without contacting nerve endings, thereby minimizing or avoiding pain [19,100,101,102,103].

### 2.7. In Vitro Release Study

**Figure 7:**
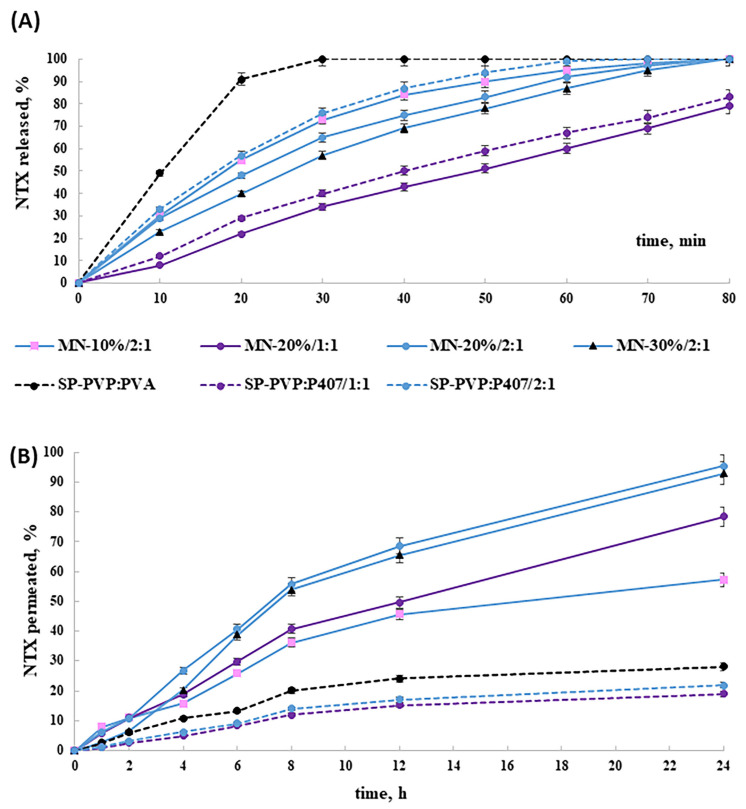
In vitro release profiles of naltrexone hydrochloride (NTX) from the prepared dissolving microneedle (DMN) and SP patch formulations (**A**), and corresponding in vitro permeation profiles of NTX from the same formulations (**B**). The results are presented as mean ± SD (n = 3).

The in vitro release profiles of the developed formulations demonstrated a clear influence of polymer composition and physicochemical properties on the dissolution behaviour of naltrexone hydrochloride (Figure 7). Since NTX is a highly water-soluble compound, the release rate from the investigated systems should be primarily governed by hydration, swelling, and dissolution of the polymer matrices rather than by drug solubility limitations.

The results demonstrated that the statistically significant differences (* *p* < 0.05) in the release behaviour of NTX from the prepared systems were mainly governed by the PVP to Poloxamer 407 ratio. The rapid release (almost 100% within 30 min) observed for the matrix patches composed of PVP and PVA can be explained by the polymers’ hydrophilic nature and high aqueous solubility. PVP readily dissolves in aqueous media, leading to rapid matrix disintegration and formation of aqueous channels that facilitate drug diffusion. Upon contact with the dissolution medium, PVA first undergoes hydration and swelling, followed by gradual dissolution. The combination of these two polymers, therefore, resulted in rapid hydration of the matrix, followed by progressive erosion, which enabled fast diffusion of the incorporated API. Consequently, the PVP/PVA matrix patches demonstrated the fastest release rate among the investigated systems.

In contrast, the patches in which the NTX was incorporated into the PVP/P407 matrix (both DMN and SP formulations) exhibited slower drug release than the PVP/PVA systems (between 34% and 76% NTX release up to 30 min). Poloxamer 407 is an amphiphilic triblock copolymer composed of polyethylene oxide and polypropylene oxide segments, which is known for its thermoresponsive behavior. Upon contact with a dissolution medium, at temperatures approaching physiological conditions (approximately 37 °C), poloxamer solutions undergo micellization followed by the formation of a viscous gel-like structure. This thermosensitive gelation increases the viscosity of the hydrated matrix and can create a temporary diffusion barrier for drug molecules. The formation of a hydrated polymer network likely prolonged the diffusion pathway of the drug, resulting in a more gradual release profile [50,67,104,105,106,107]. However, as the matrix hydrated and polymer chains dispersed, the local concentration of poloxamer decreased below the critical gelation threshold, resulting in gradual erosion and complete dissolution of the film [68,108].

These findings suggest that the physicochemical properties of the polymers, particularly their hydration rate, viscosity development, and dissolution characteristics, strongly influence drug liberation from the matrix. Moreover, since polymer hydration determines the formation of aqueous diffusion pathways within the matrix, the solubility of the polymeric components is also expected to affect the subsequent permeability and diffusion of the drug.

The microneedle-based patch formulations exhibited similar release behaviour with their corresponding simple matrix patches. In these systems, NTX was incorporated into the backing layer composed of Poloxamer 407 and PVP, while the MN tips consisted of PVP and PVA. Upon contact with the dissolution medium, the hydrophilic PVP/PVA tips rapidly hydrated and dissolved, allowing the medium to penetrate the backing layer and promote drug release. However, since the drug was not located directly in the microneedle tips, the release process required hydration of the backing layer, which delayed the onset of drug diffusion compared with the rapidly dissolving PVP/PVA matrix patches (34% NTX released up to 30 min for MN-20%/1:1 compared to 40% for SP-PVP:P407/1:1).

Even though not as significant, the polymer concentration in the microneedle tips also influences NTX release from the prepared DMN patches. Increasing the polymer concentration in the tips from 10% to 30% resulted in progressively slower drug release, from 73% to 57% up to 30 min. Higher polymer content generally produces a denser polymeric network with reduced porosity and slower hydration, thereby delaying the penetration of the dissolution medium into the microneedle structure.

The obtained results indicate that the dissolution behaviour of the investigated systems is strongly influenced by the solubility and hydration properties of the polymers used in the formulations. Highly water-soluble polymers, such as PVP, promoted rapid matrix erosion and fast drug release, whereas polymers capable of forming viscous or gel-like structures, such as Poloxamer 407, tended to slow release by creating diffusion barriers. These polymer-related effects are further expected to influence subsequent permeation behaviour, since the rate of NTX liberation from the formulation determines the concentration gradient driving diffusion across the membrane. Consequently, formulations with faster polymer dissolution are likely to generate higher drug availability at the membrane surface, which may enhance the overall permeation rate.

### 2.8. In Vitro Permeability Study

In vitro permeation studies are a critical step in evaluating topical and transdermal drug delivery systems, including microneedle (DMN) patches, as they provide essential information on the API’s ability to cross the skin barrier and reach the systemic circulation or deeper skin layers. Such studies allow for the assessment of formulation performance, drug release from the dosage form, and the influence of excipients on drug transport under controlled experimental conditions. For this purpose, synthetic membranes are often used as alternatives to biological tissues to reduce variability and improve experimental reproducibility. In the present study, permeation experiments were performed using the Strat-M membrane, a multilayered artificial membrane designed to mimic the structural and diffusional characteristics of human skin. This membrane consists of a lipid-treated dense surface layer supported by porous polymeric layers that simulate the barrier properties of the stratum corneum and deeper skin regions. Compared with excised human or animal skin, Strat-M offers several practical advantages, including minimal batch-to-batch variability, improved safety, easier handling, and long-term storage stability, while still providing permeation profiles that correlate well with those obtained from biological membranes. These characteristics make Strat-M a reliable and widely used model for the preliminary screening and comparative evaluation of transdermal formulations such as microneedle patches [109,110,111,112].

The obtained permeation profiles (Figure 7) demonstrated clear differences (* *p* < 0.05) between conventional matrix patches and microneedle-assisted systems, highlighting the critical role of microneedles in enhancing transmembrane drug delivery. All simple matrix patch formulations exhibited relatively low and insufficient permeation throughout the 24 h experiment (under 24% for 12 h), confirming the limited diffusion of NTX across the membrane when the skin barrier is not mechanically disrupted [113]. However, a noticeable difference between the polymer matrices was observed. The patch composed of the PVP:PVA (SP-PVP:PVA) blend demonstrated higher permeation (24% for 12 h) compared with the PVP:P407 formulations (15% for the same time interval). Similar to the observation already made from the in vitro dissolution studies, this behaviour could be attributed to the physicochemical characteristics and the nature of polymers [114].

**Figure 8:**
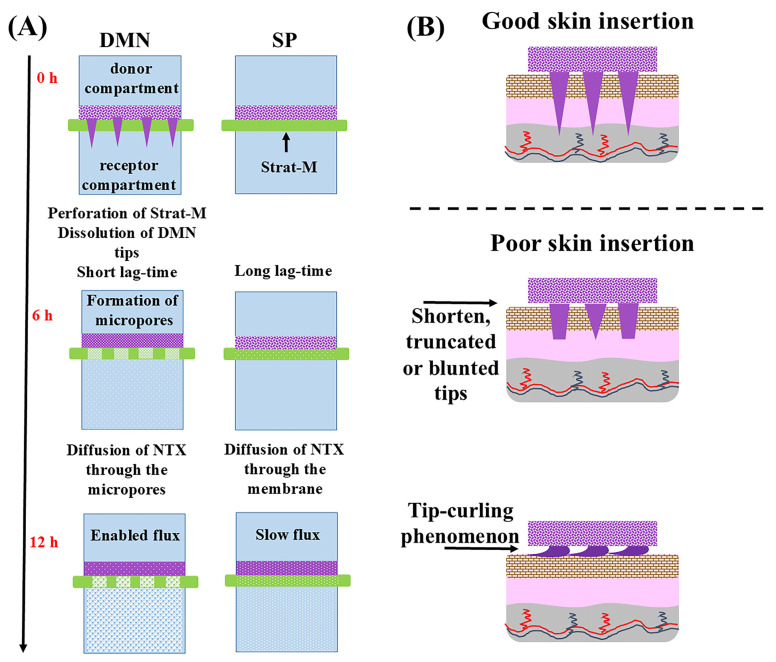
A schematic representation of the permeation mechanism of NTX from dissolving microneedle (DMN) and SP patch formulations, illustrating direct drug delivery through microchannels created after microneedle dissolution versus passive diffusion across the stratum corneum from SP patches (**A**), and in illustration of good versus poor microneedle skin insertion, comparing complete penetration of the stratum corneum with inadequate insertion leading to reduced drug delivery efficiency (**B**).

Compared with simple matrix patches, all DMN patch formulations demonstrated markedly enhanced permeation, confirming microneedles’ ability to overcome the primary barrier function of the stratum corneum. NTX permeation from MN-20%/2:1 at 12 h was 68%, which is a 4-fold increase compared to its corresponding matrix patch (SP-PVP:P407/2:1) at the same time point. By creating microscopic channels across the membrane, the microneedles provide direct pathways for drug diffusion, significantly reducing the diffusional resistance of the barrier layer. Once the membrane in vitro (or stratum corneum in vivo) is disrupted, hydrophilic drugs can rapidly diffuse through the formed pores [115]. As a result, the cumulative amount of permeated drug from microneedle patches was substantially higher than that observed for the conventional patches during the entire experimental period [113,116,117,118]. Despite the improved permeation provided by DMN patches, the permeation profiles displayed a short lag phase (2–4 h) during the initial stage of the experiment. The observed lag-time before the steady-state flux is a common event for DMN formulations and depends on the formulation and pore formation kinetics [117,119]. This phenomenon in the prepared systems can be attributed to their design, in which the API was incorporated into the backing layer (not the microneedle tips). Upon application, the microneedle tips rapidly dissolve upon contact with the aqueous medium, creating microchannels within the membrane [29]. However, since the drug is located in the backing layer, an additional diffusion step is required for the drug molecules to migrate through the hydrated polymer matrix and reach the microchannels. This results in the observed lag time before significant permeation occurs (Figure 8A).

The composition of the microneedle tips also influenced the permeation behaviour. Except for formulation MN-10%/2:1, the observations are consistent with the results obtained from the in vitro release studies, where higher polymer concentrations in the tips may lead to higher viscosity, slower dissolution, and slightly delayed pore formation, which can moderate the early permeation rate [114]. Nevertheless, once the microneedles were dissolved and the microchannels were established, the influence of the tip composition became less significant. Despite the observed faster in vitro dissolution rate of NTX from formulation MN-10%/2:1, the in vitro permeation rate through Strat-M membrane was significantly low (45% for 12 h), compared to the other DMN patches (68% and 65% for 12 h). This could be attributed to the lower insertion ability and mechanical strength determined for this formulation. A lower polymer concentration was reflected in the production of mechanically shorter, truncated and brittle tips with poor penetration efficiency, preventing the formation of sufficient microchannels in the Strat-M membrane. Strat-M thickness (300 µm) is higher than the deepest insertion layer that can be reached by the MN-10%/2:1 patch, making insertion inefficient (Figure 8B). These findings indicated that drug permeation in microneedle systems was primarily governed by insertion efficiency rather than release rate alone. Despite the faster release profile, insufficient penetration and limited microchannel formation significantly restricted overall drug transport across the membrane.

At later stages of the experiment, the permeation behavior was predominantly governed by the composition of the backing layer, which serves as the drug reservoir. Differences between formulations with varying polymer ratios in the backing layer were therefore observed. Systems containing higher proportions of poloxamer showed significantly reduced permeation compared with those containing lower amounts of this polymer (49% versus 68% for 12 h). This can be explained by the thermosensitive behavior of poloxamer, which leads to the formation of a more viscous hydrated matrix at physiological temperature, thereby slowing drug diffusion through the backing layer. Besides this, another possible reason for the unsatisfactory permeation behaviour of composition MN-20%/1:1 could be the observed lower toughness, determined for the backing layer of this formulation, compromising adequate support insertion during microneedle patch application. In contrast, formulations with higher PVP proportions (MN-20%/2:1) facilitated faster polymer hydration and dissolution, resulting in enhanced drug transport. Thus, the backing-layer composition is of high importance, influencing insertion, diffusion and permeation [76]. These findings highlight the importance of optimizing both structural components of the microneedle patch in order to achieve an optimal balance between rapid channel formation and controlled drug release from the polymeric reservoir. Thus, the overall performance of the system is determined by the combined effect of microneedle tip properties, which govern insertion and microchannel formation, and backing-layer characteristics, which control drug release and subsequent diffusion through the created pathways.

### 2.9. Differential Scanning Calorimetry

**Figure 9:**
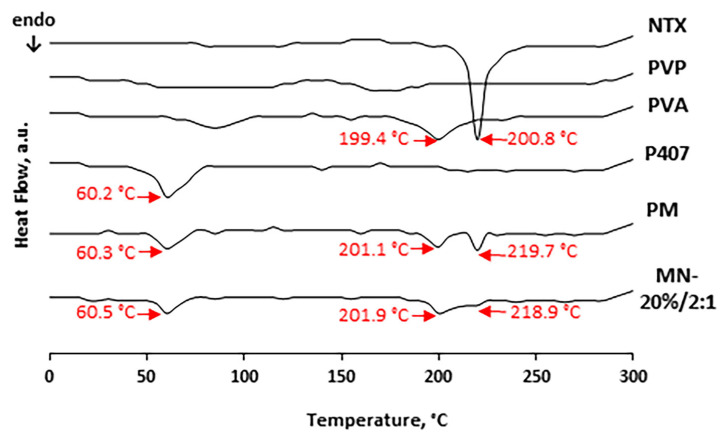
Differential scanning calorimetry (DSC) thermograms of NTX, individual excipients (polyvinylpyrrolidone, PVP; polyvinyl alcohol, PVA; and Poloxamer 407, P407), their physical mixture (PM), and the optimized microneedle formulation MN-20%/2:1, used to evaluate the thermal behavior and potential interactions between components.

Differential scanning calorimetry (DSC) analysis was performed to investigate the thermal behavior of NTX, the selected polymers, their physical mixture (PM), and the optimized microneedle formulation (MN-20%/2:1), as well as to evaluate possible physicochemical interactions between the drug and excipients. The obtained thermograms are presented in Figure 9.

Pure NTX exhibited a characteristic sharp endothermic peak at 219.8 °C, corresponding to its melting temperature and confirming its crystalline nature [120]. Similar thermal behavior of NTX has been previously reported in the literature. In contrast, PVP demonstrated a broad and diffuse thermal profile without a distinct melting endotherm, which is consistent with its amorphous structure and hygroscopic nature. A small broad endothermic event observed at lower temperatures may be attributed to the evaporation of absorbed moisture [121]. PVA exhibited a broad endothermic transition within the range of approximately 190–210 °C, corresponding to the melting of its semicrystalline domains [122,123,124]. Poloxamer 407 showed a characteristic low-temperature endothermic transition around 50–70 °C associated with its melting and micellization behavior [125].

The thermogram of the physical mixture (PM) retained the characteristic thermal transitions of the individual components. The NTX melting endotherm was reduced in intensity compared with pure NTX, likely due to the relatively low concentration of NTX compared with the polymer components. Importantly, no additional peaks or major thermal shifts indicating chemical incompatibility were observed.

Similarly, the optimized microneedle formulation (MN-20%/2:1) demonstrated substantial reduction and broadening of the characteristic NTX melting peak, overlapping with the PVA endotherm. This may be attributed to homogeneous dispersion of the drug within the polymeric network during the solvent-casting and drying processes, which may contribute to improved drug release and permeation behavior [47,120]. The absence of new thermal events further supports the compatibility of NTX with PVP, PVA, and Poloxamer 407 in the developed formulation.

### 2.10. Stability Study

Stability evaluation was performed for the optimized microneedle formulation MN-20%/2:1 in order to assess the potential influence of environmental conditions, considering humidity, on the mechanical integrity of the system during storage. This formulation was selected for the stability study because it demonstrated the most favourable balance between mechanical performance and permeation behaviour. The parameters monitored during storage were the percentage reduction in microneedle height after compression testing, Parafilm penetration ability and API assay [126,127].

The samples were stored at 25 °C under two controlled relative humidity conditions, namely 43% RH and 86% RH. These environments were chosen to simulate moderate and high humidity conditions that may be encountered during the storage and handling of microneedle patches. Humidity is a critical factor for polymeric dissolving microneedles because the hydrophilic polymers commonly used in their composition can absorb moisture from the surrounding environment. Water uptake may lead to polymer plasticization, increased chain mobility, and a consequent reduction in mechanical stiffness, which could negatively affect their mechanical properties [127,128].

The microneedle tips in the optimized formulation are composed of a blend of PVP and PVA. PVP is known to be highly hygroscopic due to the presence of lactam groups capable of forming hydrogen bonds with water molecules, which makes it particularly susceptible to humidity-induced plasticization [94,129]. In contrast, PVA possesses a partially crystalline structure that provides improved mechanical rigidity and can partially counteract the softening effect caused by moisture uptake. The backing layer of the system contains a blend of PVP and Poloxamer 407. Poloxamer 407 is less hygroscopic in the solid state and generally exhibits good physical stability [130].

**Figure 10:**
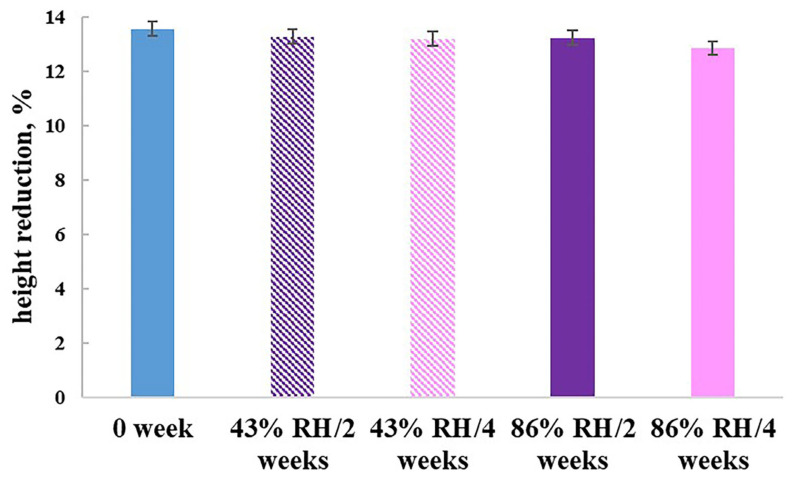
Percentage reduction in microneedle height (%) of the MN-20%/2:1 patch formulation under different relative humidity conditions over storage periods of 0, 2, and 4 weeks, presented as mean ± SD (n = 3).

**Figure 11:**
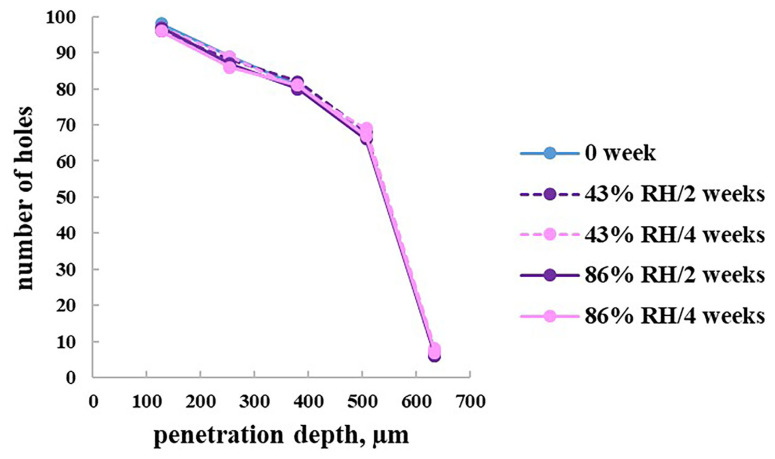
Parafilm M penetration test of the MN-20%/2:1 patch formulation, expressed as the number of microchannels (holes) formed per Parafilm layer, evaluated under different relative humidity conditions over storage periods of 0, 2, and 4 weeks, presented as mean ± SD (n = 3).

The obtained results (Figure 10) demonstrated that the percentage reduction in microneedle height remained relatively constant throughout the four-week storage period under both humidity conditions. At 43% RH, the height-reduction values varied only slightly from 13.57% at week 0 to 13.21% at week 4. Similarly, under the more stressful condition of 86% RH, the values ranged from 13.57% initially to 12.87% after four weeks. These minor fluctuations are very small and fall within the typical experimental variability of mechanical testing. Considering penetration ability (Figure 11), the formulation MN-20%/2:1 demonstrated no statistically significant changes (* *p* > 0.05) in the insertion efficiency upon storage for four weeks at either humidity level.

The results demonstrate that the optimized MN-20%/2:1 formulation maintained stable mechanical characteristics during storage under both humidity conditions. The statistically insignificant changes (* *p* > 0.05) in the percentage height reduction and insertion efficiency confirmed that the microneedle structure remains mechanically robust, suggesting that the formulation possesses adequate physical stability for further development and evaluation [97,101].

The assay of API from MN-20%/2:1 patch formulation, stored at different humidity levels, did not show any statistically significant changes (* *p* > 0.05), compared with the initial value, again suggesting sufficient stability.

## 3. Materials and Methods

### 3.1. Materials

Naltrexone hydrochloride (NTX), supplied by INCHEM LABORATORIES PVT. Ltd. (Hyderabad, India), was used as a model drug. Polyvinylpyrrolidone (PVP) with a molecular weight (Mr) of 58 kDa and Poloxamer 407 (Pluronic^®^ F127) were obtained from Sigma Aldrich Chemie GmbH (Steinheim, Germany). Polyvinyl alcohol (PVA) with Mr 30 kDa was purchased from Fluka Chemie GmbH (Buchs, Switzerland). Strat-M^®^ membranes were obtained from Merck Millipore (Darmstadt, Germany). All other reagents and solvents were of analytical grade and used as received. Deionized water was used for all preparations.

### 3.2. Fourier-Transform Infrared (FTIR) Spectroscopy

Fourier-transform infrared (FTIR) spectroscopy was employed to observe the potential physicochemical interactions between naltrexone hydrochloride and the selected excipients—PVP, PVA and Poloxamer 407. Spectral analysis was performed using a Nicolet Impact 400D FT-IR spectrometer (Thermo Scientific Nicolet, Waltham, MA, USA). Spectra were acquired within the wavenumber range of 4000–400 cm^−1^ at a spectral resolution of 4 cm^−1^. Binary physical mixtures were prepared by mixing pure NTX with each excipient at ratios corresponding to those employed in the respective model formulations. The prepared mixtures were transferred into 5 mL glass vials, hermetically sealed, and stored in a thermostatic chamber at 60 °C for 15 days. After storage, FTIR spectra were recorded and compared with those of the individual components to detect potential shifts, disappearances, or emergences of characteristic absorption bands.

### 3.3. Preparation of Dissolving Microneedle Patches (DMN Patches)

**Figure 12:**
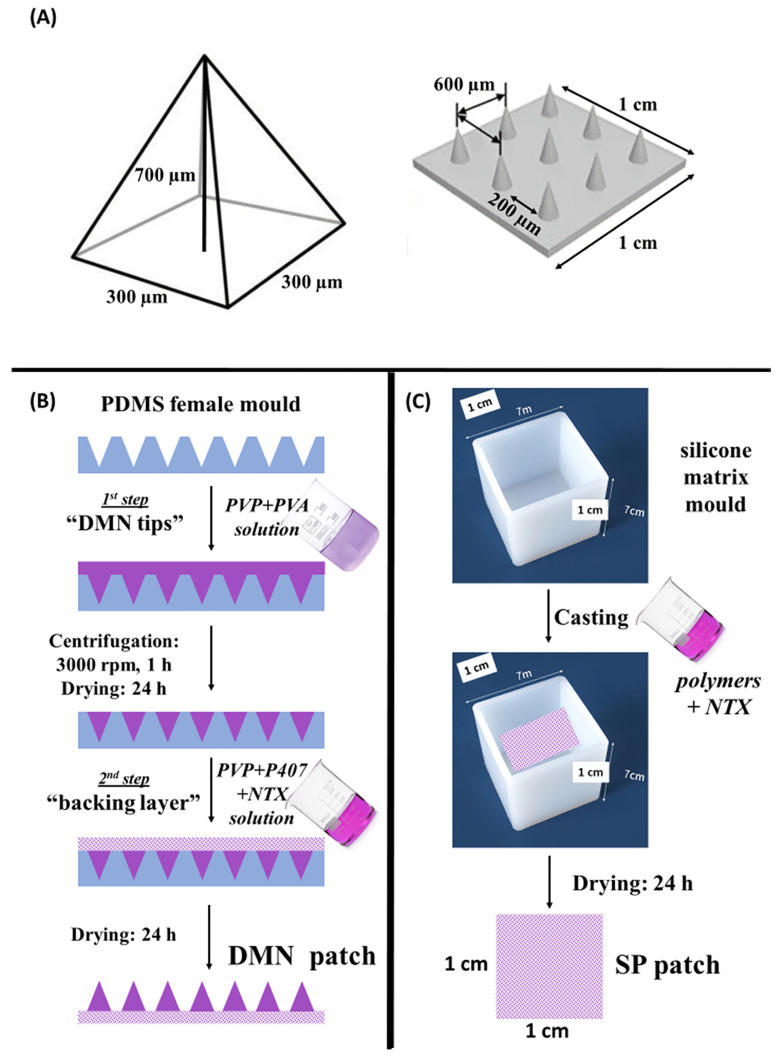
A schematic representation of microneedle tip geometry illustrating the general structural features of the microneedle array (**A**), the fabrication process of dissolving microneedle (DMN) patches using a two-step micromoulding technique (**B**), and the preparation of SP patches via a solvent casting method (**C**).

Dissolving microneedle patches were prepared using a two-step micromoulding technique, including tip and backing-layer casting, as shown in Figure 12B. Microneedle tip formulations contained polymer blends of polyvinylpyrrolidone (PVP, Mw 58 kDa) and polyvinyl alcohol (PVA, Mw 30 kDa) at total solid concentrations of 10%, 20% or 30% and at a weight ratio of 2:1. Initially, polymer solutions were prepared separately under continuous magnetic stirring—PVA were dissolved in purified water by heating at 80 °C and PVP in purified water at room temperature. The obtained polymer solutions were then mixed in the appropriate ratio and allowed to stand to eliminate entrapped air bubbles prior to casting. The technological procedure continued, pouring the solutions into female 10 × 10 polydimethylsiloxane (PDMS) microneedle moulds (area of 1 cm^2^) consisting of 100 pyramidal tips—700 μm in height, with 300 μm width at the base and 200 μm interspacing (Figure 12A). Centrifugation was carried out at 3000 rpm for 30 min in both directions, then complete filling of the mould cavities was ensured. The residual polymer solution remaining on the mould surface after filling was carefully removed using a spatula. The moulds were subsequently dried at room temperature in a desiccator for 24 h until complete solidification of the microneedle tips was achieved.

The backing-layer formulations were prepared at a total polymer blend concentration of 30% (*w*/*w*), including Poloxamer 407 and PVP (Mw 58 kDa) at two weight ratios—1:1 and 2:1. Poloxamer 407 was dissolved in cold water (4 °C) under gentle stirring and stored in the refrigerator. The corresponding amounts of PVP and naltrexone hydrochloride (0.1% *w*/*w*, relative to the total formulation) were subsequently added to the clear poloxamer solution under continuous stirring until a homogeneous solution was obtained. Following complete drying of the microneedle tips, 5 mL of the backing-layer formulation was cast over the moulds containing the preformed needles. The systems were allowed to dry under controlled conditions (in a desiccator at room temperature) for 24 h until a uniform backing layer was formed. After complete drying, the obtained dissolving microneedle patches were carefully removed from the moulds and stored at room temperature.

### 3.4. Preparation of Simple Matrix Patches (SP Patches)

Simple matrix-type transdermal patches (SP) containing 0.1% (*w*/*w*) naltrexone hydrochloride (NTX) were prepared by solvent casting using three different 30% (*w*/*w*) total solid polymer blends, as expressed in Figure 12C. The formulation SP-PVP:PVA contained a mixture of PVP (Mw 58 kDa) and PVA (Mw 30 kDa) in a weight ratio of 2:1. Formulations SP-PVP:P407/1:1 and SP-PVP:P407/2:1 represented a polymer blend of PVP (Mw 58 kDa) and Poloxamer 407 in a 1:1 and 2:1 weight ratio, respectively. The polymer blend solutions were prepared similarly to those described in the previous section. All formulations were degassed, and 5 mL of each was cast onto 1 cm^2^ PDMS flat moulds to obtain uniform films, which were dried under controlled conditions (in a desiccator at room temperature) for 24 h until complete solvent evaporation. The dried films were carefully removed from the moulds and stored at room temperature.

### 3.5. Scanning Electron Microscopy (SEM) of DMN Patches

The micromorphology of the prepared DMN patch formulations was studied via a scanning electron microscope (JSM-5510, JEOL, Akishima, Japan) operating at an accelerating voltage of 10 kV. The samples were coated with gold for 30 s using a sputter-coater (JSC 1200, JEOL, Japan) in an inert argon atmosphere before imaging.

### 3.6. The API Assay

The NTX assay in each formulation (DMN and SP patch) was determined by immersing a 1 cm × 1 cm patch sample in 25 mL of purified water under magnetic stirring for 3 h, followed by filtration through a 0.45 µm syringe filter. The drug content was analysed spectrophotometrically at λ = 280 nm (Thermo Scientific Evolution 300, Madison, WI, USA) and quantified against a standard calibration curve of NTX. The percentage of the API amount per one patch was calculated via Equation (1) [97]. The experiment was performed in triplicate and expressed as mean ± standard deviation (SD).

(1) $$API\;assay,\%=\frac{API\;amount\;in\;the\;sample\times 100}{theoretical\;API\;amount}$$

### 3.7. In Vitro Gelation Temperature and Gelling Time and Rheology

The thermoresponsive behavior of the poloxamer-containing systems was characterized for gelation temperature and in vitro gelling time. Four aqueous solutions corresponding to backing-layer formulations were prepared, containing 30% total polymer blend of PVP and Poloxamer 407 at ratios of 2:1 and 1:1, with and without 0.1% naltrexone hydrochloride. The gelation temperature was determined via the utilization of a modified tube inversion approach [131]. Briefly, 10 g of each formulation was transferred into a glass vial and placed in a temperature-controlled paraffin bath. The samples were heated gradually at a rate of approximately 1 °C per minute under continuous stirring using a magnetic stirrer set at 175 rpm (IKA RCT standard, IKA-Werke GmbH & Co.KG, Staufen, Germany). The temperature was monitored using a calibrated digital thermometer (IKA ETS-D5, IKA-Werke GmbH & Co.KG, Staufen, Germany). The gelation temperature was determined as the temperature point at which the magnetic stir bar ceased moving, corresponding to the transition from a liquid to a gel state. The in vitro gelling time was evaluated in a phosphate-buffered solution media (pH 7.4). All samples were stored at room temperature for 6 h prior to testing. An aliquot (100 µL) of each formulation was carefully poured into 2 mL of pre-heated buffer maintained at 35 ± 0.5 °C. Gelling time, expressed as loss of fluidity, was determined visually [132]. All experiments were conducted in triplicate [131].

The temperature-dependent rheological behavior of the prepared formulations was characterized using a HAAKE RheoStress 600 rheometer (Thermo Scientific, Karlsruhe, Germany). Measurements were carried out using a parallel plate geometry (20 mm diameter) with a gap distance of 1 mm. The samples were analyzed over a temperature range of 10–50 °C with a controlled heating rate of 1 °C/min. Oscillatory measurements were performed in deformation-controlled mode at a constant frequency of 1 Hz and a strain amplitude of 1%. During the analysis, the storage modulus (G′), loss modulus (G″), and viscosity (η) were continuously monitored as a function of temperature to evaluate the thermoresponsive characteristics of the formulations. The gelation temperature was determined from the rheological profiles as the temperature corresponding to the transition from viscous-dominant to elastic-dominant behavior, identified by the crossover of G′ and G″ values [72,74,75,133]. All measurements were conducted in triplicate.

### 3.8. Mechanical Properties

#### 3.8.1. Three-Point Bend Testing of Simple Matrix Patches

Three-point bending tests were performed to evaluate the mechanical properties of the simple matrix patches, containing 30% polymer blends of PVP:Poloxamer 407 (1:1 and 2:1) and PVP:PVA (2:1), corresponding to DMN backing-layer compositions. The study was conducted using a texture analyzer (TA-XT Plus, Stable Micro Systems, Godalming, UK) equipped with a three-point bending rig. The test specimens were cut from the dried films and stored at room temperature for at least 24 h prior to testing. Each sample was placed on two parallel supports of the bending rig with a span distance of 4.8 mm, ensuring it was properly positioned between the supports. The probe was aligned above the midpoint between the supports and moved vertically downward to apply a bending force to the film. The instrument was equipped with a 10 kg load cell. The trigger force was adjusted to 25 g, providing consistent contact between the probe and the film surface. During the test, the applied mechanical force (g) and probe displacement (mm) were recorded in order to generate force–displacement curves. The obtained data were used to determine the mechanical characteristics of the films, including hardness, flexibility, and toughness [76,134,135]. All measurements were performed in triplicate for each formulation, and the results were expressed as mean ± SD.

#### 3.8.2. Mechanical Strength of DMN Patches (Fracture Force Test)

**Figure 13:**
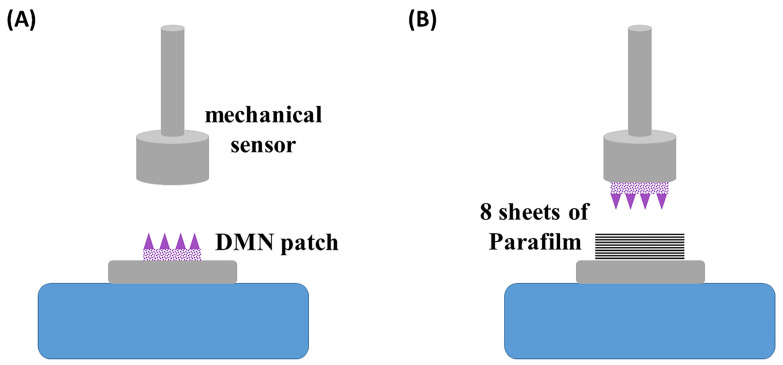
A schematic representation of the mechanical evaluation of dissolving microneedle (DMN) patches using a texture analyzer (TA-XT Plus, Stable Micro Systems, UK), illustrating the compression force (fracture force) test setup in which axial load is applied to the microneedle array (**A**), and the Parafilm M penetration test configuration used to assess insertion performance by quantifying the number of microchannels formed across successive Parafilm layers (**B**).

Mechanical testing of the microneedle arrays was performed to evaluate their structural integrity and suitability for skin insertion. To characterize the resistance of the obtained DMN patches to compressive forces, a compression analysis was carried out using a texture analyzer (TA-XT Plus, Stable Micro Systems, UK) operating in compression mode, as demonstrated in Figure 13A. For the measurements, each DMN patch was fixed to the cylindrical probe using double-sided adhesive tape, ensuring the tips were aligned with the direction of the applied force. A compressive force was then applied with a maximum load of 32 N—corresponding to the average force exerted by a human thumb [96,136], simulating realistic conditions of use. The microneedle tips were examined under a digital light microscope (Light microscope Leica DM750, Heerbrugg, Switzerland) before and after compression to evaluate structural integrity and further deformation. To quantify deformation following compression, the height of the tips was measured before (H1) and after (H2) the compression test and the percentage reduction in microneedle height was calculated according to the following equation, Equation (2):

(2) $$Height\;reduction,\%=\frac{\left(H1-H2\right)\times 100}{H1}$$

#### 3.8.3. Parafilm Penetration Test of DMN Patches

The insertion efficiency of the prepared DMN patches was evaluated using an eight-layer Parafilm^®^ M as an elastic skin-mimicking model [52,96,101,136]. The DMN arrays were positioned on top of the Parafilm sheets, and insertion was performed using a Texture Analyzer (TA-XT Plus, Stable Micro Systems, UK) under the same conditions as the fracture force test (Figure 13B). Following insertion, the DMN were removed, and the Parafilm sheets were observed under a digital light microscope to assess penetration depth. The pores formed in each Parafilm layer were quantified, and the penetration ability was expressed using Equation (3) [128,137].

(3) $$Penetration\;of\;n\;layer=\frac{number\;of\;holes\;in\;n\;layer\times 100}{total\;number\;of\;holes}$$

### 3.9. In Vitro Drug Release Study

A modified paddle-over-disk-type dissolution method was employed using an incubator shaker (Julabo Shake Temp SW23, Seelbach, Germany) to determine the release of NTX from both DMN and SP patch formulations. The patches were affixed to a stainless-steel disk assembly and placed at the bottom of the vessels containing 50 mL phosphate-buffered saline (pH 7.4), which were maintained at 32 ± 0.5 °C and 50 rpm shaking rate. At predetermined intervals (5, 10, 15, 20, 30, 45, and 60 min), 1 mL samples were withdrawn and immediately replaced with an equivalent volume of fresh preheated PBS to maintain constant volume and sink conditions. The collected samples were analyzed spectrophotometrically at 280 nm against blank solution. All measurements were performed in triplicate, and the results are expressed as mean ± SD. [138,139].

### 3.10. In Vitro Permeability Study

The in vitro permeation of NTX from the developed DMN patches and matrix patches was evaluated in accordance with the recommendations of the European Medicines Agency for transdermal drug delivery systems [140]. The experiments were performed using an automated transdermal diffusion cell sampling system, Logan System 913-6 (Logan Instruments Corp., Somerset, NJ, USA). A synthetic membrane (Strat-M membrane) was used as a barrier separating the donor and receptor compartments [112,141,142]. The membrane was mounted between the two compartments of a Franz diffusion cell, providing an effective diffusion area of 1.54 cm^2^. DMN patches (1 cm^2^) as well as matrix patches, containing an equivalent amount of approximately 5 mg NTX, were placed in the donor compartment and applied onto the membrane with gentle pressure to ensure proper contact. To guarantee the proper holding of the patches on the membrane, a 5 g weight was placed on top. Phosphate-buffered solution (pH 7.4) with a volume of 12 mL was used as the receptor medium. The diffusion cells were maintained at 32 ± 0.5 °C under continuous stirring at 100 rpm throughout the whole 24 h experiment. At predetermined time intervals, 1 mL samples were automatically withdrawn from the receptor compartment and immediately replaced with an equal volume of fresh buffer to maintain sink conditions. The amount of permeated drug was quantified spectrophotometrically at a wavelength of 280 nm. All experiments were performed in triplicate and the results are expressed as mean ± SD.

The cumulative amount of drug permeated through the membrane per unit surface area (Q_t_, µg/cm^2^) was calculated using the following equation, Equation (4) [47,97,137,143,144]:

(4) $$Q_{t}=\frac{C_{n}.V_{A}+\sum_{i}^{n-1}C_{i}.V_{s}}{A}$$

where Q_t_ represents the cumulative NTX amount penetrated through the membrane; C_n_—NTX concentration in the nth sample; Ci—NTX concentration in the ith sample; V_A_—acceptor medium volume (12 mL); V_s_—sample volume (1 mL); and A—effective permeation surface area of the membrane (1.54 cm^2^).

The percentage of the API permeated relative to the initially applied dose was determined using the following Equation (5) [145]:

(5) $$API\;permeated,\%=\frac{Qt\times 100}{M}$$

where Qt represents the cumulative amount of naltrexone hydrochloride permeated through the membrane at time t (µg/cm^2^), and M is the total amount of the API initially loaded in the tested patch (µg/cm^2^).

### 3.11. Differential Scanning Calorimetry (DSC)

The thermal properties of pure NTX, the selected polymers PVP, PVA, P407, and their physical mixture (PM), as well as the optimized formulation (MN-20%/2:1), were evaluated using the differential scanning calorimeter PerkinElmer DSC-8500 (Waltham, MA, USA), equipped with an Intracooler 3. The respective samples were loaded into standard aluminum pans and then scanned. The temperature range covers the diapason from −50 °C to 300 °C with a heat rate of 10 °C/min. Pyris software v.10.1.0.0412 was used for device control and data collection.

### 3.12. Stability Study

Stability studies of the chosen DMN formulation were conducted under controlled environmental conditions at 25 °C and two different relative humidity levels. Relative humidity of 43% was maintained using a saturated potassium carbonate solution, while a saturated potassium chloride solution was used to generate a relative humidity of 86% [94,97,146]. The representative DMN patch samples were stored under these conditions in airtight containers for a period of one month. Following storage, the mechanical characteristics of the DMN patches and the API assay were evaluated and compared with the initial values in order to assess potential changes during storage.

### 3.13. Statistical Analysis

Statistical evaluation of the obtained data was carried out using one-way analysis of variance (ANOVA) followed by Tukey’s multiple-comparison test employing GraphPad (GraphPad Software, San Diego, CA, USA). Experimental results were expressed as mean ± standard deviation (SD) based on three independent measurements. Different asterisks indicate statistically significant differences between formulations, where * *p* < 0.05, ** *p* < 0.01, and *** *p* < 0.001. Statistical differences between groups were considered significant at *p* < 0.05.

## 4. Conclusions

The present study demonstrated the successful design and development of dissolving microneedle patches with an advanced architecture for transdermal delivery of naltrexone hydrochloride. The combination of PVP/PVA-based microneedle tips and a PVP/Poloxamer 407 backing layer proved to be an effective strategy for balancing mechanical performance and drug delivery characteristics. The selected polymer blend for the tips ensured adequate mechanical strength for insertion while maintaining suitable dissolution behavior, whereas the thermoresponsive backing layer functioned as a drug reservoir capable of modulating release and permeation.

The results clearly indicated that polymer concentration plays a critical role in microneedle formation and performance. Both low and high polymer concentrations resulted in suboptimal microneedle morphology and reduced tip height due to insufficient mechanical integrity or hindered mould filling, respectively. In contrast, the intermediate polymer concentration (20%) provided optimal viscosity, enabling efficient cavity filling, reduced shrinkage, and formation of well-defined microneedles with superior mechanical properties and insertion ability. Mechanical testing and insertion studies confirmed that the optimized formulation (MN-20%/2:1) exhibited the most favorable balance between strength, flexibility, and penetration efficiency. Furthermore, in vitro release and permeation studies demonstrated that microneedle-assisted delivery significantly enhanced naltrexone transport compared with conventional matrix patches, confirming the system’s ability to overcome the skin’s barrier function. The study also demonstrated the importance of the composition of the backing layer for the overall patch performance. The incorporation of NTX only in the backing layer enabled high, reproducible, and uniform drug loading, without compromising tip mechanical integrity, whereas the proper PVP:Poloxamer 407 ratio contributed to a more controlled drug release profile due to its thermoresponsive gel-forming behaviour.

Overall, the developed microneedle system could be considered a promising platform for transdermal delivery of NTX, offering improved permeation, controlled release, and patient-friendly administration. The findings highlight the importance of formulation optimization, particularly polymer composition and concentration, in achieving an effective and reliable microneedle-based drug delivery system.

## 5. Limitations

This study has several limitations that should be considered when interpreting the results. First, no ex vivo or in vivo studies were conducted due to ethical and institutional constraints. Therefore, the performance of the microneedle systems was evaluated only using in vitro models, including the Strat-M membrane. Although this model is widely used, it does not fully replicate the complexity of biological skin, and the correlation between in vitro findings and in vivo performance remains to be established. Further ex vivo and in vivo studies are needed to confirm the effectiveness, safety and therapeutic relevance of the system, especially for supporting the hypothesis that in situ gelation of poloxamer could contribute to preventing micropore closure in the skin following microneedle dissolution.

Another limitation concerns the scalability of the fabrication process. The microneedles were produced using a laboratory-scale micromoulding method, which may present challenges in large-scale manufacturing. Factors such as reproducibility, uniform filling of moulds, drying conditions, and batch-to-batch consistency may vary under industrial conditions.

In addition, only a limited range of polymer concentrations and formulation parameters was investigated, and further optimization may be possible. These aspects should be addressed in future work to support the translation of the developed microneedle system.

## Figures and Tables

**Table 1:** Composition of the prepared DMN and SP patches.

DMN Patches				SP Patches		
MN-10%/2:1	MN-20%/1:1	MN-20%/2:1	MN-30%/2:1	SP-PVP:PVA	SP-PVP:P407/1:1	SP-PVP:P407/2:1
**DMN tips**				30% PVP:PVA = 2:1	30% PVP:P407 = 1:1	30% PVP:P407 = 2:1
PVP:PVA = 2:1						
10%	20%	30%	30%			
**DMN backing layer**				0.1% NTX		
0.1% NTX						
30% PVP:P407						
2:1	1:1	2:1	2:1			

**Table 2:** API (naltrexone hydrochloride) assay (%) per one patch, mean ± SD (n = 3).

Formulation Code	API Assay, %
MN-10%/2:1	97 ± 4
MN-20%/1:1	98 ± 3
MN-20%/2:1	98 ± 4
MN-30%/2:1	98 ± 4
SP-PVP:PVA	98 ± 3
SP-PVP:P407/1:1	98 ± 3
SP-PVP:P407/2:1	96 ± 2

**Table 3:** Gelation temperature and gelling time of the prepared solutions upon buffer media with pH 7.4, mean ± SD (n = 3).

SP-PVP:P407/2:1	SP-PVP:P407/1:1	SP-PVP:P407/2:1—API Free	SP-PVP:P407/2:1—API Free
**gelation temperature, °C**			
34.2 ± 0.2	31.6 ± 0.3	33.9 ± 0.2	30.8 ± 0.5
**gelling time, s**			
17.5 ± 0.6	9.17 ± 0.15	15.97 ± 0.17	8.3 ± 0.2

**Table 4:** Height reduction (%) of the prepared DMN patch formulations determined via mechanical strength test, mean ± SD (n = 3).

Parameter	MN-10%/2:1	MN-20%/1:1	MN-20%/2:1	MN-30%/2:1
**H1, µm**	540 ± 40	610 ± 60	641 ± 18	550 ± 30
**H2, µm**	270 ± 30	510 ± 40	554 ± 17	4 70 ± 30
**height reduction, %**	50 ± 3	18 ± 5	13.6 ± 1.4	15 ± 3

## Data Availability

Data are available from the authors (see the email addresses provided).

## Abbreviations

The following abbreviations are used in this manuscript:

API	Active pharmaceutical ingredient
DMN	Dissolving microneedles
DMN patch	Dissolving microneedle patch
DSC	Differential Scanning Calorimetry
FT-IR	Fourier Transform Infrared
MN	Microneedles
NTX	Naltrexone hydrochloride
P407	Poloxamer 407
PNIPAM	Poly(N-isopropylacrylamide)
PVA	Polyvinyl alcohol
PVP	Polyvinylpyrrolidone
SD	Standard deviation
SEM	Scanning electron microscopy
